# Better Outcomes in Severe and Morbid Obese Patients (BMI > 35 kg/m^2^) in Primary Endo-Model Rotating-Hinge Total Knee Arthroplasty

**DOI:** 10.1100/2012/249391

**Published:** 2012-04-30

**Authors:** Luis M. Lozano, Vicente López, José Ríos, Dragos Popescu, Pere Torner, Félix Castillo, Francisco Maculé

**Affiliations:** ^1^Knee Surgery Unit, Orthopaedic Department, Hospital Clínic, Universitat de Barcelona, 08036 Barcelona, Spain; ^2^Orthopaedic Department, Hospital Dos de Maig, 08025 Barcelona, Spain; ^3^Laboratory of Biostatistics & Epidemiology, Universitat Autonoma de Barcelona and IDIBAPS, 08036 Barcelona, Spain; ^4^Orthopaedic Department, Consorci Sanitari Parc Taulí, 08208 Sabadell, Spain

## Abstract

The Endo-Model rotating-hinge prosthesis is preferably indicated as a primary implant in patients with advanced axial deviation of the lower limbs or unstable knees with severe bone defects. Outcomes were studied in 111 knees, operated in a three-year period; the mean followup was 28 months. Joint balance enhancement and limbs mechanical axis correction were achieved after surgery. There were 6 deep infections and 16 patients referred postoperative anterior knee pain. WOMAC index scores disaggregated by gender and BMI showed better outcomes in obese patients (specifically, those with a BMI of 35–40 kg/m^2^) and in men. Although the lack of a control group did not allow definite conclusions and despite a nonnegligible complication rate, our results reveal that the Endo-Model total knee arthroplasty can be a useful tool to deal with severe and morbid obese patients affected of severe gonarthrosis associated with marked axial deviations, ligament instability, or bone defects.

## 1. Introduction

Constrained rotating-hinge knee prostheses are a useful orthopaedic tool in the treatment of advanced tibiofemoral deformities and severe bone and ligament defects [1–3]. The new rotating models attempt to avoid torsional stress which produces premature loosening, and to reduce the patellofemoral tension, characteristic of nonrotating constrained prostheses [1, 2]. Few studies have been published [4–9] regarding the primary indication of the Endo-Model rotating-hinge implant (Waldemar Link GmbH & Co), with most research focusing on its use in revision surgery [3–5, 9–12]. The previous generation of this implant was associated with several complications, being patellofemoral pain one of the most frequent [2, 6–9, 12]. Actual implant includes an anterior femoral shield with the aim to solve this problem. This paper reports the outcomes obtained and the complications which arose when using the Endo-Model rotating-hinge implant for primary total knee replacement.

## 2. Patients and Methods

A descriptive observational study with prospective followup was performed over a three-year period, from January 2006 to January 2009. During this time 120 primary cemented Endo-Model rotating hinge prostheses (Waldemar Link GmbH & Co) were implanted in patients with degenerative knee joint disease. A total of nine prostheses were excluded from the final analysis: six were lost to followup and there were three exitus. Minimum follow-up time established was one year. The mean admission time was 6.1 days, while the mean follow-up time in the series was 28 months [17; 36].

The Endo-Model is a cemented rotating knee prosthesis with a long stem and a central hinge system that enables controlled flexion and rotation, which can be converted intraoperatively into a constrained system with an antiluxation device. The model includes an anterior femoral shield, which, in theory, minimizes the mechanical tension that can arise in the patellofemoral compartment.

The study included 111 surgical prostheses (14 bilateral). Gender distribution was 84 women and 27 men, with a mean age of 72.77 years. The mean BMI was 30.81 kg/m^2^ (median: 31.1 [27.3; 33.3]). As regards the preoperative mechanical axis 51 patients (46%) presented a valgus deformity and 60 (54%) varus deformity (Table 1).

In 21 knees radiological study revealed prentervention bone defects. Physical examination of the knees prior to surgery showed instability in 38 cases (34.2%). The preoperative range of movement was 94.55° of flexion [65; 130] and −3.51° of extension [−20; 0]. The median preoperative tibiofemoral mechanical axis was 10° [−14°; 20°]. The distribution of ASA anaesthetic risk was six cases (5.4%) ASA I, 84 cases (75.7%) ASA II, and 21 cases (18.9%) ASA III.

Indications were (1) varus or valgus deformities associated with ligament instability and/or fixed flexion deformity greater than 15° or bone defects without extensor apparatus damage; (2) soft tissue retraction that would require complete release of the lateral ligaments; (3) knees that were stable but with varus deviations of more than 20° or valgus deviations of more than 14°.

The variables analyzed in order to assess outcomes were complications, patellofemoral pain, joint balance and limb alignment after surgery, and changes in the patient's functional status, measured according to the WOMAC (Western Ontario and McMaster Universities) osteoarthritis index [5–13]. The WOMAC index comprises 24 items that are grouped into three categories: pain, stiffness, and physical function. It was considered to be the most suitable instrument for determining any postintervention changes in patients functioning. The WOMAC questionnaire was completed prior to surgery and again at the last follow-up assessment (which must be at least one year after surgery). Scores on the WOMAC index were categorized as follows: >38: poor outcome; 29–38: acceptable outcome; 15–28: good outcome; and 0–14: excellent outcome [13].

The results obtained were analyzed according to the following factors: body mass index (BMI) (the cutoff point for obesity being a BMI ≥ 30 kg/m^2^ [14]), the presence of associated comorbidity measured by the ASA scale for anaesthetic risk [15], the diagnosis regarding the joint degeneration, and the presence of postoperative complications. These factors were considered in order to characterize the patients and, when necessary, to determine whether one or more of them had influenced the outcomes obtained.

Surgical difficulty was evaluated on the basis of ischemia time, measured from inflation of the tourniquet to its release once the implant was cemented. Perioperative complications and surgical difficulties were recorded in the surgical log at the end of the intervention. Patellar alignment was studied by using the axial at a 45° angle projection (skyline patella view), measuring both the patellar tilt with respect to the femoral component and the lateral patellar displacement. Deep infection was considered when positive intra-articular cultures were obtained. An infection was defined as acute if it was diagnosed in the first six weeks after surgery [16]. Active mobilization was started on the first day after surgery, with early weight bearing of the affected limb being introduced from day two.

**Statistical Analysis**

The results are presented as means and standard deviations (SD) or medians with the 25th and 75th percentiles [P25; P75] for quantitative variables and as absolute frequencies and percentages for qualitative data. Potential prognostic factors related to changes in the WOMAC index were evaluated by means of ANCOVA models which included, in addition to the preoperative WOMAC score, defining variables of the pathology (etiology and diagnosis) and demographic variables that could be useful in defining a specific target population susceptible to the use of this prosthesis (in this case, age, sex, and obesity, the latter defined as a BMI ≥ 30 kg/m^2^). The remaining associations were analyzed in the usual way by means of Fisher's exact test for qualitative data and the Mann-Whitney *U* test for quantitative variables. Within-patient postintervention changes in knee mobility were assessed using McNemar's test. In all tests, the two-sided Type I error rate was set at 5% and SPSS v. 15 was used to perform all the analyses.

## 3. Results

Three patients (2.7%) suffered complications during surgery, consisting in two fractures of the medial femoral condyle (which were fixed with Kirschner wires) and one case of cement leaking into the anterior femoral cortex. The median ischemia time (measured to assess surgical difficulty) was 65 minutes (range: 32–120 min). The mean blood loss, measured as the amount of blood collected from the two drains 48 hours after surgery, was 463.4 cc [250; 850]. A deep venous thrombosis (DVT) was diagnosed by doppler ultrasound and resolved with medical treatment.

There were six acute deep infections (5.4%). The relationship between infection and variables as BMI, ASA, duration of surgery, and the presence of diabetes mellitus were studied, but founded no significant in any case.

There were sixteen (14.4%) cases of postoperative patellofemoral pain. Of these group, eleven (68.8%) also presented an alteration (subluxation and patella tilting) on the radiographic images obtained in the axial plane (skyline view), whereas among those without pain (95 patients) radiographic changes were only observed in ten (10.5%) cases. A statistically significant relationship between the presence of patellofemoral pain and radiographic evidence of axial deviation was found. (Fisher's exact test, *P* < 0.001).

**Figure 1:**
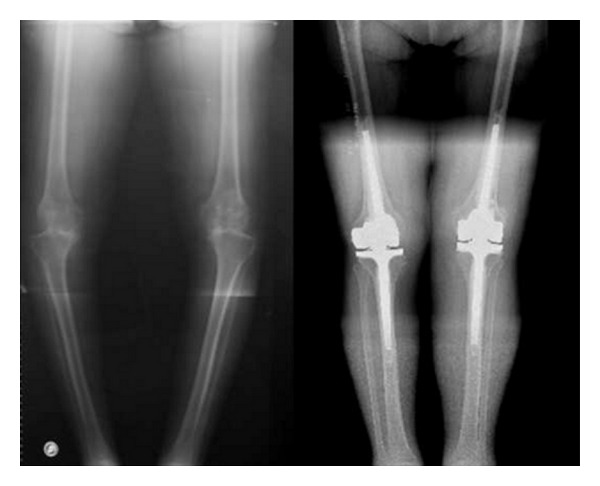
Preoperative X-ray of a patient with a severe bilateral varus deformity. Postoperative image of bilateral prosthesis showing the axial correction.

The second variable studied was improvement in joint balance and limb mechanical axis correction after surgery. As regards the active range of motion the median preoperative flexion was 90° [90°; 100°], while the median flexion at followup was 120° [100°; 120°]. With respect to extension, 33 (30%) knees showed negative extension (fixed flexion deformity) preoperatively (range: −5 to −30°), of which 30 cases (90%) achieved 0° (normal) extension after the intervention. Two knees (2.6%) of the 77 (70%) cases with normal preoperative extension (0°) showed a fixed flexion deformity at followup (McNemar's test, *P* < 0.001; Table 2). In all cases it was possible to correct the mechanical axis (Figure 1).

Clinical status was evaluated by calculating changes in WOMAC scores at followup. Scores on the WOMAC scale fell (indicating improved functional status) 35.67 points (SD: 20.43). Furthermore, the initial WOMAC score was found to be significantly correlated (*P* < 0.001) with the observed changes: the higher the initial WOMAC score (i.e., worse preoperative functional status), the greater the subsequent difference. BMI was also found to be related to changes in the WOMAC score. Patients with a BMI ≥ 30 improved their WOMAC score by 6.9 points (95% CI: 0.3; 13.4). Men improved their WOMAC score by 9.2 points (95% CI: 1.7; 16.7) more than women. However, etiology was not a significant factor in terms of explaining changes in the WOMAC score (*P* = 0.712) (Table 3). 

Although the presence of knee pain had no significant influence on differences in the total WOMAC score (*P* = 0.079), patients who did not report such pain improved their WOMAC score by −7.9 (95% CI: −16.7; 0.93) with respect to those with anterior knee pain. As regards the three WOMAC dimensions, the greatest differences in improvement were observed on the pain (−2.8, 95% CI: −4.3; −0.24; *P* = 0.029) and stiffness (−0.71, 95% CI: −1.41; −0.011; *P* = 0.046) dimensions, in both cases favoring those patients without patellofemoral pain. The greatest change on the WOMAC index corresponded to patients with a BMI of 35–40 kg/m^2^, who showed a statistically significant improvement of −46.06 points (95% CI: −58.92; −33.20; *P* < 0.003). The change in obese patients was statistically significant with respect to both nonobese (BMI < 25 kg/m^2^) and overweight (BMI 25–29.99 kg/m^2^) patients.

## 4. Discussion

Endo-Model is a long cemented stem prosthesis with excellent primary stability and adequate diaphyseal fixation. The traditional indications are nonreducible varus deformities of more than 16° and nonreducible valgus deformities of more than 12°, as well as ligament instability and bone defects [1–3, 8, 17].

Perioperative complications encountered in the present study are similar to those reported when using constrained rotating-hinge prostheses [7, 12]. Fractures of the femoral condyles were resolved with simple osteosynthesis without underlying consequences. Fractures of the medial condyle occur performing the femoral cuts and adapting the central cage of the femoral component.

Postoperative infection is a common complication in this arthroplasty, whether used for primary or revision surgery [4, 6, 8, 18]. The high rate of acute postoperative infections observed (5.4%) could be attributable to the preoperative health status [8]. However, there was no statistically significant relationship between infection and the presence of associated comorbidity (diabetes), the ASA score, the ischemia time, or BMI. The rate of deep vein thrombosis and pulmonary thromboembolism was in line with reported in other series [7, 12].

**Figure 2:**
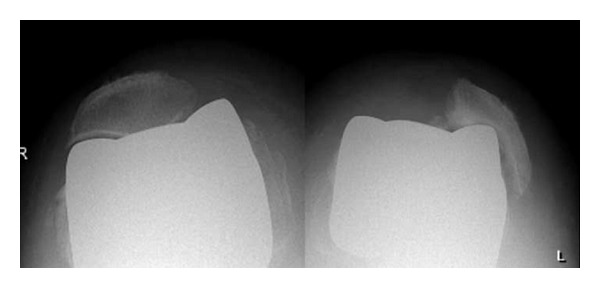
Bilateral image showing a patellofemoral deformity with obvious patellar luxation at the left side.

Extensor apparatus realignment is a factor to be considered when using this prosthetic model [2–4, 7, 8]. In this regard, a high number of patients (14.4%) presented patellofemoral problems at followup and a statistically significant relationship between the presence of patellofemoral pain and radiographic evidence of axial patelar deviation was found. Previous studies have reported an incidence of anterior knee pain from 5 to 22%, depending on the series [4, 7, 9]. Argenson and Aubaniac [8] reported patellar problems in 5% of patients fitted with the Endo-Model rotating-hinge prosthesis and attributed these problem to the lack of patellar resurfacing and insufficient release of the lateral patellar retinaculum. Petrou et al. [7] studied the relationship between stair-climbing difficulty and patellar alignment in the axial plane, reporting that patients who were unable to climb stairs showed a grade-IV misalignment (patellar shift and tilt). The present data are in line with these findings, since misalignment of the patella was observed in patients who reported anterior knee pain after the intervention (Figure 2). It should be noted, however, that the prosthesis used here (which includes an anterior femoral shield to facilitate the fit and alignment of the patella) was different from that described in the study by Petrou et al. [7]. Given the present results, it seems that this modification does not avoid this frequent complication.

Although some authors argue that the subvastus approach and release of the lateral patellar retinaculum are possible solutions to this problem [7, 8] we believe that incorrect rotation of the tibial and femoral components should be considered as the cause of patellofemoral misalignment [6].

A femoral component with internal rotation orientation will facilitate patella tilting and persistent external subluxation. We recommend in cases of marked varus deformity, to perform the tibial entry point more external than surgeons are used to. In cases of genu valgus the entry point must be situated at the centre of the tibial spines. The use of computed axial tomography to study the implant orientation could shed light on this problem [19–22].

Postoperative WOMAC index assessment revealed significant improvements related to preoperative status. Patients with the greatest functional limitations prior to surgery improved more than others with less functional limitations. This improvement was independent of their age and the etiology of their joint degeneration, even though some authors have reported worse outcomes in patients with inflammatory disease [7]. 

Gender and BMI influenced the outcomes obtained: the greatest improvement (46 points) was observed among obese patients with a BMI of 35–40 kg/m^2^. Given the increasing number of obese patients with gonarthrosis that requires surgical treatment the present data suggest that the prosthetic model used should be considered when the degree of axial deviation or knee instability makes it advisable. It should be noted, however, that a survival study by Katzer et al. [6], found that obesity was a predisposing factor for prosthetic loosening, although they considered weight rather than BMI when analysing the results of their series. As regards gender, the prosthesis used led to better functional outcomes in men (Table 3). The lack of male patients in some of the BMI categories made it impossible to determine whether the better outcomes for men compared to women were maintained across the entire range of BMI. It would be necessary to study whether this difference is related to the social role of men, which is not differentiated by the WOMAC index.

The relatively short follow-up period in the present series (mean of 28 months) may constitute one of the major limitations of the study, since it prevents long-term outcomes from being assessed and implant survival curves from being plotted. A larger sample would also enable the inclusion of more men, thus increasing the likelihood of obtaining more conclusive results regarding the relationship between postoperative improvement, gender, and BMI. On the other hand, the present series constitute a homogenous and representative sample of the population in our catchment area and was recruited during a short period of time, thus increasing the internal validity of the study.

In conclusion, the considerable postoperative improvements reflected by the WOMAC index may justify the inclusion of this prosthesis, as a primary indication, in the orthopedic therapeutic arsenal to deal with selected severe and morbid obese patients affected of severe gonarthrosis with marked axial deviations, ligament instability, or bone defects. Furthermore, the Endo-Model-rotating hinge prosthesis requires a precise surgical technique in order to avoid postoperative patellofemoral problems.

## Figures and Tables

**Table 1:** Baseline data. Results are expressed as mean (SD) for quantitative data and as *n* (%) for qualitative data.

		*N* = 111
Sex	Female	84 (75.7%)
	Male	27 (24.3%)
Age		72.77 (8.89)
Diagnosis	Valgus	51 (45.9%)
	Varus	60 (54.1%)
Aetiology	Osteoarthritis	93 (83.8%)
	Rheumatoid arthritis	11 (9.9%)
	Posttraumatic arthropathy	7 (6.3%)
Preop. WOMAC-Total		59.35 (15.95)

**Table 2:** Preoperative extension versus postoperative extension.

		Postaoperative extension	
		0°	Fixed flexion
Preoperative extension	0°	75 (97.4%)	2 (2.6%)
	Fixed flexion	30 (90.9%)	3 (9.1%)

McNemar, *P* < 0.001			

**Table 3:** Changes in WOMAC score adjusted for preoperative status and patient age for each of the factors studied. Results are expressed as mean and 95% confidence interval. Age was not statistically significant in the fitted model (*P* = 0.097). The initial WOMAC score was statistically significant in the fitted model (*P* < 0.001).

BMI	<30	≥30	

(*P* = 0.04) WOMAC	−32.1 (−39.5; 24.7)	−38.9 (−46.4; −31.6)	
		Differences	*P*
		6,9 (0.3; 13.4)	0.04

Gender	Women	Men	

(*P* = 0.017) WOMAC	−30.9 (−37.4; 24.5)	−40.1 (−48.8; −31.5)	
		Differences	*P*
		9.2 (1.7; 16.7)	0.017
